# FLT3 inhibitors in acute myeloid leukemia

**DOI:** 10.1186/s13045-018-0675-4

**Published:** 2018-12-04

**Authors:** Mei Wu, Chuntuan Li, Xiongpeng Zhu

**Affiliations:** 1 Department of Hematology, The People’s Hospital of Bozhou, Bozhou, 236800 China; 2 Department of Hematology, First Hospital of Quanzhou affiliated to Fujian Medical University, Quanzhou, 362000 China

**Keywords:** FMS-like tyrosine kinase 3 inhibitors, Acute myeloid leukemia, Midostaurin, FLT3

## Abstract

FLT3 mutations are one of the most common findings in acute myeloid leukemia (AML). FLT3 inhibitors have been in active clinical development. Midostaurin as the first-in-class FLT3 inhibitor has been approved for treatment of patients with FLT3-mutated AML. In this review, we summarized the preclinical and clinical studies on new FLT3 inhibitors, including sorafenib, lestaurtinib, sunitinib, tandutinib, quizartinib, midostaurin, gilteritinib, crenolanib, cabozantinib, Sel24-B489, G-749, AMG 925, TTT-3002, and FF-10101. New generation FLT3 inhibitors and combination therapies may overcome resistance to first-generation agents.

## Introduction

Acute myeloid leukemia (AML) remains a highly resistant disease to conventional chemotherapy, with a median survival of only 4 months for relapsed and/or refractory disease [1]. Molecular profiling by PCR and next-generation sequencing has revealed a variety of recurrent gene mutations [2–4]. New agents are rapidly emerging as targeted therapy for high-risk AML [5, 6]. In 1996, FMS-like tyrosine kinase 3/internal tandem duplication (FLT3/ITD) was first recognized as a frequently mutated gene in AML [7]. According to 2017 ELN risk stratification, patients with FLT3/ITD^high^-positive AML are classified into adverse risk category. This mutation causes resistance to conventional chemotherapy. Although patients with AML can be cured with hematopoietic stem cell transplantation (HSCT), most of these patients are at high risk for relapse. Thus, the overall cure rate of AML is only 30–40% [1].

FLT3/ITD gene is found in approximately 30% of patients with AML with normal cytogenetics. FLT3/ITD belongs to the type III family of receptor tyrosine kinases [8]. The FLT3 gene is located on chromosome 13 .q12. It is expressed mainly in human hematopoietic progenitors and dendritic cells and plays key roles in leukemia cell proliferation, differentiation, and survival [9]. Constitutive activation of the FLT3/ITD gene triggers multiple downstream signaling cascades, such as STAT5, RAS, MEK, and PI3K/AKT pathways [10], and ultimately causes suppression of apoptosis and differentiation of leukemic cells, including dysregulation of leukemic cell proliferation [11].

Multiple FLT3 inhibitors are in clinical trials for treating patients with FLT3/ITD-mutated AML. In this review, we summarized the preclinical and clinical studies on new FLT3 inhibitors, including sorafenib, lestaurtinib, sunitinib, tandutinib, quizartinib, midostaurin, gilteritinib, crenolanib, cabozantinib, Sel24-B489, G-749, AMG 925, TTT-3002, and FF-10101.

### First-generation FLT3 inhibitors

#### Sorafenib

Sorafenib is an oral multikinase inhibitor of RAF-1, VEGF, c-KIT, PDGFR, ERK, and FLT3. Currently, sorafenib is approved for treating hepatocellular carcinoma and renal cell carcinoma. Sorafenib also has a potent anti-leukemic effect on FLT3-mutated AML. It completely inhibits FLT3/ITD activity with an IC_50_ of 69.3 ng/ml [12].

#### Mechanisms of sorafenib effects on AML

There are several mechanisms that explain the efficacy of sorafenib for treating AML. First, sorafenib promotes secretion of IL-15 by FLT3/ITD-mutated leukemic cells and improves the survival time of patients with FLT3/ITD-positive AML [13]. Secondly, persistently low blast percentage, CD3^+^ cell invasion in the epidermis, high proportion of CD8^+^ lymphocytes in the bone marrow, and high expression levels of COL4A3, TLR9, FGF1, and IL-12 genes have been observed in patients treated with sorafenib [14]. Sorafenib has also been shown to block Src kinase-mediated STAT3 phosphorylation and reduces expression of apoptosis regulatory proteins such as Mcl-1 and Bcl-2 [15]. Finally, sorafenib decreases Smac mimetic-induced necroptosis in apoptosis-resistant leukemia cells [16].

#### Sorafenib plus chemotherapy for treating AML

Sorafenib combined with conventional chemotherapy is being explored for AML therapy. Sorafenib and decitabine have been used in preclinical and clinical trials to treat FLT3/ITD-mutated AML in vitro and in vivo [17]. Both drugs showed synergistic anti-tumor effects in a human FLT3/ITD-mutated AML cell line. In a clinical study, 5 out of 6 patients showed overall favorable responses, including 4 relapsed/refractory patients achieving complete remission with incomplete count recovery (CRi). The median survival time of these patients was 155 days, and the drugs were well tolerated. Ravandi et al. reported the effects of sorafenib combined with 5-azacytidine (AZA) in 43 patients with AML, including 40 patients with FLT3/ITD mutation [18]. All patients were intravenously administered AZA 75 mg/m^2^/d for 7 days and orally administrated sorafenib 400 mg continuously. Response rate (RR) was 46%, complete remission with incomplete count recovery (CRi) 27%, complete remission (CR) 16%, and partial remission (PR) 3%. Recently, Mahdi et al. used the same doses of azacytidine and sorafenib to successfully treat a pregnant patient with FLT3/ITD-mutated AML [19]. After 1 cycle of azacytidine and sorafenib treatment, the number of blasts in the bone marrow markedly decreased and FLT3/ITD was undetectable. The patient was also independent of transfusion, and her neutrophil count was almost normal after 4 cycles of treatment. Most importantly, the newborn was healthy. Sorafenib was also evaluated in a multicenter single-arm phase II study in patients aged ≥ 60 years with FLT3-mutated AML (Table 1) [20]. Sorafenib was added to induction, consolidation, and maintenance therapies. Fifty-four patients were enrolled in the study, 39 of which were FLT3/ITD-positive. The 1-year overall survival (OS) in FLT3/ITD patients was 62%, and disease-free survival (DFS) and OS were 12.2 and 15.0 months, respectively. In another multicenter, randomized, double-blind, placebo-controlled phase 2 trial from Germany, 267 patients with AML were treated with sorafenib and standard chemotherapy [21]. The result from this study showed that sorafenib had prolonged event-free survival (EFS), but the toxicity was also increased.

**Table 1:** Clinical trials of sorafenib for AML therapy

Study agents	Other agents	Disease	No Pts	Response	Reference
Sorafenib	Decitabine	Relapsed Refractory	6	OR 83% CR 80%	[17]
Sorafenib	5-azacytidine	Relapsed	43	OR 46%	[18]
Sorafenib	Daunorubicin Cytarabine	Untreated	54	1-year OS 62% DFS 12.2 months OS 15 months	[20]
Sorafenib	Daunorubicin Cytarabine	Untreated	267	EFS 21 months 3-year EFS 40%	[21]

*OR* overall response, *CR* complete remission, *OS* overall survival, *DFS* disease-free survival, *EFS* event-free survival

#### Application of sorafenib in HSCT

Sorafenib has shown encouraging results in HSCT for patients with FLT3/ITD-positive AML. In a retrospective analysis, 17 patients with FLT3/ITD-positive AML received sorafenib in combination with allo-HSCT [22]. Among the 17 patients, 10 patients started sorafenib only after transplantation. Fourteen of the 17 patients achieved CR, whereas 5 patients eventually progressed. Five patients showed pronounced signs of toxicity but remained in complete molecular remission when the dosage schedule was alternated. Sorafenib combined with allo-HSCT induced a lower relapse rate and longer leukemia-free survival (LFS) in patients with FLT3/ITD-mutated AML. In another study, 144 patients treated with the same regime were divided into 4 groups. The 3-year relapse rate of the four groups was 22.2%, 18.8%, 15.8%, and 46.1%, whereas OS and LFS rates were 74.9%, 78.1%, 84.6%, and 50.9% and 69.4%, 78.1%, 80.4%, and 34.8%, respectively [23]. Brunner et al. examined the effect of sorafenib as a maintenance drug for patients with FLT3/ITD-mutated AML in the first complete remission after HSCT [24]. The 2-year OS and PFS in the 26 sorafenib-treated patients were 81% and 82%, respectively. The 2-year cumulative incidence of relapse was 8.2%. However, there was no difference in 2-year non-relapse mortality or 1-year cGVHD between the sorafenib-treated patients and control. In another study of sorafenib as a maintenance drug after HSCT, 27 pediatric patients with FLT3/ITD-positive AML were enrolled [25]. Of these, 25 patients achieved complete molecular remission. The 1-year OS and PFS were 92 ± 6% and 92 ± 5%, respectively. Sorafenib was also used as a salvage therapy pre- and post-transplantation for 16 patients with refractory/relapsed FLT3-ITD-positive AML (Table 2) [26]. Out of the 16 patients, 13 achieved CR. The 2-year OS and DFS were 75.0 ± 10.8% and 50.5 ± 13.7%, respectively. Skin rash and gastrointestinal and cardiac toxicities were observed. In a report of a long-term follow-up of 29 patients with relapsed FLT3/ITD-positive AML after allo-SCT and sorafenib treatment [27], the median follow-up was 7.5 years. In this report, 6 patients survived, with 5 patients achieving sustained complete remission and 4 patients in treatment-free remission for a median of 4.4 years.

**Table 2:** Clinical trials of sorafenib in hematopoietic stem cell transplantation

Study agents	Other agents	Disease	No Pts	Response	Reference
Sorafenib	Allo-HSCT		17	CR 82%	[22]
Sorafenib	Allo-HSCT		144	3-year OS 74.9%/78.1%/84.6%/50.9% 3-year relapse rates 22.2%/18.8%/15.8%/46.1% 3-year LFS rates 69.4%/78.1%/80.4%/ 34.8%	[23]
Sorafenib	Maintenance		81	2-year OS 81% 2-year PFS 82% 2-year relapse incidence 8.2%	[24]
Sorafenib	Maintenance		27	1-year OS 92 ± 6% 1-year PFS 92 ± 5%	[25]
Sorafenib	Chemotherapy Monotherapy	Relapsed	16	CR 81%	[26]

*CR* complete remission, *OS* overall survival, *PFS* progression-free survival, *allo-HSCT* allogeneic hematopoietic stem cell transplantation, *LFS* leukemia-free survival

#### Sunitinib

Sunitinib (SU11248) is a small-molecule FLT3 inhibitor with selectivity for PDGFR, VEGFR1, VEGFR2, KIT, and FLT3 [28]. It has both direct anti-tumor and antiangiogenic properties. The use of sunitinib is currently approved for treating renal cell carcinoma, gastrointestinal stromal tumor, and AML.

#### Mechanisms of sunitinib on AML

The mechanism of sunitinib’s effect against AML is similar to that of sorafenib [29]. One study found that STAT5 phosphorylation in patients with FLT3/ITD was also reduced [30]. Intriguingly, SU11248 shows synergistic effects with cytarabine or daunorubicin in inhibiting proliferation and survival of primary AML myeloblasts expressing mutant FLT3/ITD, FLT3/D835V, or FLT3/WT [31]. Furthermore, sunitinib induces G1 phase arrest, increases pro-apoptotic molecule expression, and decreases anti-apoptotic molecule expression in AML cells [32].

#### Sunitinib combined with chemotherapy for AML

In the past few years, more clinical trials of sunitinib with chemotherapy have been conducted. In a phase I/II clinical trial, sunitinib and intensive chemotherapy were chosen for 22 patients with FLT3/ITD-mutated AML aged over 60 years [33]. Thirteen patients, including 8 patients with FLT3/ITD mutation, achieved CR/CRi. The median overall, relapse-free, and event-free survival of the 17 patients were 1.6, 1.0, and 0.4 years, respectively. In another phase I study, 15 patients with refractory AML were treated with SU11248 [34]. Patients with FLT3 mutations showed morphologic or partial responses. No dose-limiting toxicity was observed in patients treated with SU11248 at 50 mg. The most common grade 2 toxicities were edema, fatigue, and oral ulcerations.

#### Lestaurtinib

Lestaurtinib (CEP-701) is an orally bioavailable indolocarbazole alkaloid compound derived from the bacterial fermentation product K-252a. It has activities against tropomyosin receptor kinases, neurotrophin receptors, FLT3, and JAK2 [35–37]. Different from other class III receptor tyrosine kinases, lestaurtinib has low IC_50_ against FLT3 phosphorylation. Interestingly, lestaurtinib is cytotoxic to human AML cell lines expressing both mutant and wild-type FLT3, and it prolongs the survival of FLT3/ITD leukemia in a mouse model [36].

#### Lestaurtinib for AML

In a phase II trial, lestaurtinib was used as a monotherapy in untreated older patients with AML [38]. Lestaurtinib was administered orally at doses of 60 mg and 80 mg twice daily for 8 weeks. Blast percentages in the bone marrow and peripheral blood in 3 out of 5 patients with mutated FLT3 were reduced transiently, and periods of transfusion independence were prolonged. In another phase I/II clinical trial, 14 patients with relapsed, refractory, or poor-risk FLT3/ITD-mutated AML received lestaurtinib as a single-agent salvage therapy at doses of 60 mg twice daily [39]. Five patients showed transient clinical responses. However, Levis et al. showed that lestaurtinib treatment after chemotherapy in the first relapse did not improve response rates nor prolong survival of patients with FLT3/ITD-mutated AML [40]. Furthermore, Knapper et al. also proved that lestaurtinib and chemotherapy as first-line therapy did not prolong 5-year overall or relapse-free survival of younger patients with untreated FLT3-mutated AML from the UK AML15 and AML17 trials (Table 3) [41].

**Table 3:** Clinical trials of lestaurtinib for AML

Study agents	Other agents	Disease	Dose	No Pts	Response	Reference
Lestaurtinib	Chemotherapy	Untreated	60–80 mg twice daily	27	OR 60% in mutated FLT3 OR 23% in wild-type FLT3	[38]
Lestaurtinib		Relapsed Refractory	60 mg twice daily	14	OR 35%	[39]
Lestaurtinib	Chemotherapy	Relapsed	80 mg twice daily	224	CR/CRp 26%	[40]
Lestaurtinib	Chemotherapy	Untreated		500	5-year OS 46% 5-year RFS 40%	[41]

*OS* overall survival, *RFS* relapse-free survival, *CR* complete remission, *CRp* complete remission incomplete platelet recovery

### Tandutinib

Tandutinib (MLN518, CT53518) is a novel quinazoline-based inhibitor of the type III receptor tyrosine kinases, FLT3, PDGFR, and KIT. Tandutinib, at a concentration that does not affect normal colony formation, was shown to inhibit blast growth in patients with FLT3/ITD-positive AML [42]. Tandutinib induces apoptosis and inhibits FLT3/ITD phosphorylation, cellular proliferation, and signaling of the MAPK and PI3K pathways [43].

The clinical effect of tandutinib in patients with AML was examined in a phase I trial. Tandutinib showed anti-leukemic activity and decreased the number of blasts in the peripheral blood as well as in the bone marrow in 40 patients with AML or high-risk MDS [44]. In addition, the combination of tandutinib with standard chemotherapy regimen exerts antiproliferative and pro-apoptotic effects on FLT3/ITD-positive blasts in AML [45]. Long-term effects of tandutinib remain to be determined.

### Midostaurin

Midostaurin (CGP41251, PKC412) is a small-molecule tyrosine kinase inhibitor (TKI) and was approved by the US FDA in 2017 for the treatment of FLT3-mutated AML [46]. It has recently been approved for newly diagnosed patients with FLT3-mutated AML and advanced systemic mastocytosis.

The clinical activity of midostaurin has been investigated in multiple clinical trials. In a phase I trial, midostaurin was administered with bortezomib alone or in combination with mitoxantrone, etoposide, and cytarabine to patients with refractory or relapsed AML [47]. The overall response rate (ORR) and CR were 82.5 and 56.5%, respectively. Ramsingh et al. used various doses of midostaurin, all-trans retinoic acid, and CLAG chemotherapy to treat relapsed/refractory AML [48]. Among all patients, 22% achieved CR and 11% achieved CRi. However, Stone et al. reported that the efficacy of midostaurin improved significantly when administered in combination with a standard chemotherapy to newly diagnosed patients with AML (Table 4) [49]. The CR rate of patients treated with midostaurin at 50 mg twice daily was 80% (FLT3-mutant 92%, FLT3/WT 74%). However, the 1-year and 2-year OS of patients with FLT3-mutated AML were similar to those of patients with FLT3-WT. Furthermore, Stone recently reported that midostaurin in combination with a standard chemotherapy significantly prolonged the OS and EFS of patients with FLT3-mutated AML. The incidence of severe adverse events was not increased by the combined treatment [50].

**Table 4:** Clinical trials of midostaurin for AML therapy

Study agents	Other agents	Disease	Dose	No Pts	Response	Clinical trial	Reference
Midostaurin	Etoposide Cytarabine	Relapsed Refractory	50 mg bid	34	ORR 82.5% CR 56.5%	Phase I	[47]
Midostaurin	ATRA Chemotherapy	Relapsed Refractory	25 mg/50 mg twice daily	10	CR 22% CRi 11%	Phase I	[48]
Midostaurin	Daunorubicin Cytarabine	Untreated	50 mg twice daily	13	CR 92% 1-year OS 85%	Phase IB	[49]
					2-year OS 62%		

*OS* overall survival, *CR* complete remission, *CRi* complete remission with incomplete count recovery, *ATRA* all-trans retinoic acid

### Second-generation FLT3 inhibitors

#### Quizartinib

Quizartinib (AC220) is a selective and highly potent second-generation class III receptor TKI [51]. Quizartinib is a potent and selective FLT3 inhibitor for AML [52]. The quizartinib dosage with the highest efficacy is 1 mg/kg once a day.

The optimum dosages and efficacy of quizartinib alone and in combination with chemotherapy in patients with AML were investigated. A phase I open-label, sequential group dose-escalation trial was the first to evaluate the safety and tolerability of quizartinib in combination with chemotherapy in 19 patients newly diagnosed with AML [53]. Out of 16 patients who achieved good response, 14 achieved CR and 2 achieved a morphologic leukemia-free state. There were no apparent additional signs of toxicity. The most common grade 3 or 4 adverse events were febrile neutropenia, neutropenia, thrombocytopenia, and anemia. In another dose-escalation study, quizartinib was used as a maintenance therapy in 13 patients with FLT3/ITD-mutated AML after allo-HSCT [54]. Two patients treated with quizartinib at 40 and 60 mg/day interrupted treatment because of grade 3 gastric hemorrhage and anemia. One patient relapsed. However, there was no maximum tolerated dose (MTD), and 60 mg daily was the highest dose studied. Quizartinib has shown a strong activity in relapsed or refractory AML. Cortes et al. reported the results of a phase I trial of quizartinib in relapsed or refractory AML for the first time [55]. Out of 76 patients, 23 showed responses, with 10 achieving CR and 13 achieving PRs. The median duration of response was 13.3 weeks, and the median survival time was 14 weeks. The most common treatment-related adverse events were nausea, vomiting, and prolonged QT interval. The maximum tolerated dose (MTD) was 200 mg/day, and the dose-limiting toxicity was grade 3 QT prolongation. Cortes and Levis reported that the CR rate reached 44 to 54% in their phase II study of relapsed and refractory AML [56, 57]. Importantly, 30- or 60-mg/day quizartinib monotherapy was reported in 76 patients with relapsed/refractory FLT3/ITD-mutated AML. Composite complete remission (CRc) rates of both groups were similar to those who received higher quizartinib doses. The incidence of corrected QT interval (QTc) above 480 ms and 500 ms was also less common [58]. Quizartinib as a salvage chemotherapy has been administered to children with relapsed acute leukemia (Table 5) [59]. The responses were evaluated in 17 patients (2 CR, 1 CRp, 1 CRi, 10 SD, and 3 PD), 7 of which were FLT3/ITD-positive (1 CR, 1 CRp, 1 CRi, and 4 SD). FLT3 phosphorylation in all patients was completely inhibited with quizartinib at 60 mg/m^2^/day.

**Table 5:** Clinical trials of quizartinib for AML therapy

Study agents	Other agents	Disease	Dose	No Pts	Clinical trials	Response	Reference
Quizartinib	Chemotherapy	Untreated	40–60 mg/day	19	Phase I	OR 84%	[53]
Quizartinib		Relapsed Refractory	12–450 mg/day	76	Phase I	OR 30%	[55]
Quizartinib		Relapsed Refractory	30 mg/day 60 mg/day	76	Phase 2b	CRc 47%	[58]
Quizartinib	Cytarabine Etoposide	Relapsed	40 mg/day 60 mg/day	22	Phase I	2 CR, 1 CRp, 1 CRi, 10 SD, 3 PD	[59]

*OR* overall response, *CR* complete remission, *CRp* complete remission with incomplete platelet recovery, *CRi* complete response with incomplete neutrophil and platelet recovery, *CRc* composite complete remission, *SD* stable disease, *PD* progress disease

#### Crenolanib

Crenolanib is a potent and selective inhibitor of FLT3/WT, FLT3/ITD, FLT3-TKD, PDGFRα/β, KIT, and FLT3/D835 [60]. Crenolanib was less disruptive of erythroid colony growth, which may result in relatively less myelosuppression than that by quizartinib. Correlative data from an ongoing clinical trial showed that sufficient levels of crenolanib could inhibit both FLT3/ITD and resistant FLT3/D835 mutants in patients with AML [61]. In a phase II trial, the tolerability and efficacy of crenolanib combined with standard induction chemotherapy was examined in patients with newly diagnosed FLT3 mutant AML [62]. There were 26 patients including 19 patients with FLT3/ITD and 3 patients with FLT3/D835 mutations. Eighty-eight percent of patients achieved CR, and overall CR/CRi rate was 96%. During a median follow-up of 6 months, only 3 patients have relapsed. In the following year, the similar result was seen in crenolanib combined with 7+3 induction and high dose cytarabine consolidation in 29 patients < 60 years old with FLT3-mutated AML [63]. A head-to-head comparison with midostaurin in combination with 7+3 was planned to further evaluate the efficacy of crenolanib. In addition, crenolanib was also used in relapsed or refractory AML. Iyer et al. reported the result of 8 patients with first relapsed or primary refractory AML who received the treatment of high-dose ara-C/mitoxantrone (HAM) and crenolanib [64]. Four patients achieved CR/CRi after 1 cycle. Only 1 patient showed a transient elevation in total bilirubin. Maro et al. used salvage idarubicin and high-dose ara-C and crenolanib to treat patients with relapsed/refractory FLT3-positive AML [65]. The ORR was 36% and median OS was 259 days. No dose-limiting toxicities (DLT) were observed. Grade I GI toxicities including nausea, vomiting, diarrhea, and abdominal pain were the major non-hematological adverse events. Crenolanib was administered at 200 mg/m^2^/day 3 times a day in another single-center phase II study in 10 patients with relapsed/refractory AML who progressed after HSCT (Table 6) [66]. The ORR was 47%. Interestingly, crenolanib was recently shown to have synergistic antileukemia activity with FLT3-targeted CAR T cells [67].

**Table 6:** Clinical trials of crenolanib for AML therapy

Study agents	Other agents	Disease	Dose	No Pts	Response	Reference
Crenolanib	Cytarabine Anthracycline	Untreated	100 mg TID	25	CR 88% CR/CRi 96%	[62]
Crenolanib	Cytarabine Anthracycline	Untreated	100 mg TID	29	CR 83%	[63]
Crenolanib	Cytarabine Mitoxantrone	Relapsed Refractory	100 mg TID	8	CR 67%	[64]
Crenolanib	Idarubicin Cytarabine	Relapsed Refractory	60/80/100 mg TID	13	ORR 36%	[65]
Crenolanib		Relapsed Refractory	200 mg/m^2^/day	10	ORR 47%	[66]

*ORR* overall response rate, *CR* complete remission, *CRi* complete remission with incomplete count recovery

#### Gilteritinib

Gilteritinib (ASP2215) is a novel dual FLT3/AXL inhibitor. Gilteritinib significantly reduced the colony-forming capacity of FLT3/ITD-positive leukemia cells [68]. Gilteritinib decreases the phosphorylation levels of FLT3 and its downstream targets in cell cultures as well as in animal models. No obvious toxicity was observed [69]. Gilteritinib was well tolerated in 252 relapsed/refractory AML patients. The ORR was 40%, whereas the RR was 52% in FLT3-mutated patients at doses ≥ 80 mg/day. More than 5% of the patients experienced serious adverse events such as fever, disease progression, neutropenia, sepsis, acute renal failure, pneumonia, pyrexia, bacteremia, and respiratory failure. Grade 3 diarrhea and transaminase elevation were limited in patients administrated at a dose of above 300 mg/day [70]. In another open-label, phase 1 study, gilteritinib was also shown to be well tolerated in Japanese patients with relapsed/refractory AML. The ORR in patients with mutated FLT3 and FLT/WT was 80% and 36.4%, respectively. The most common drug-related severe adverse events were thrombocytopenia and increased creatine phosphokinase. The recommended phase II dose was 120 mg/day and MTD was 200 mg/day (Table 7) [71]. A phase III clinical trial comparing guilteritinib to a salvage chemotherapy regimen in relapsed/refractory FLT3-mutated AML patients is currently being conducted.

**Table 7:** Clinical trials of gilteritinib for AML therapy

Study agents	Disease	Dose	No Pts	Clinical trials	Response	Reference
Gilteritinib	Relapsed Refractory	20–450 mg/day	252	Phase 1/2	ORR 40% ORR(FLT^+^) 52%	[70]
Gilteritinib	Relapsed Refractory	20–300 mg/day	24	Phase 1	ORR(FLT3^+^) 80% ORR(FLT/WT^+^) 36.4%	[71]

*ORR* overall response rate

### Other FLT3 inhibitors

#### Cabozantinib

Cabozantinib is an oral inhibitor of multiple receptor tyrosine kinases VEGFR-1, VEGFR-2, VEGFR-3, Kit, MET, AXL, KIT, FLT3, and RET [72, 73]. It exhibits anti-tumor activity in several cancers, such as AML and renal cell carcinoma. Currently cabozantinib has been approved for treatment of advanced renal cell carcinoma. Cabozantinib exerts significant cytotoxicity to leukemia cell lines with FLT3/ITD. It induces apoptosis in leukemia cell by regulating the anti-apoptotic and pro-apoptotic proteins [74]. A phase I study of cabozantinib was done in 18 patients with AML [75]. Peripheral blast reductions were seen in 4 patients, 1 showed marrow blast reduction, and 1 had stable disease. The MTD of cabozantinib was 40 mg daily. The most common grade 2 or higher toxicities observed were fatigue, nausea, transaminitis, and electrolyte imbalance.

#### SEL24-B489

Sel24-B489 is a novel dual pan-PIM and FLT3**/**ITD inhibitor. SEL24-B489 suppresses the growth of AML cell lines. Unlike selective FLT3/ITD or PIM inhibitors, SEL24-B489 exhibits significantly broader on-target activity in AML cell lines, primary AML blasts, and FLT3-TKD-mutated cells [76].

#### G-749

G-749 is a novel FLT3 inhibitor against FLT3-ITD, D835Y, ITD/F691 L, and ITD/N676D. G-749 was shown to have sustained inhibition of FLT3 phosphorylation and downstream effectors in FLT3**/**ITD-positive as well as FLT3/WT- cell lines. It displayed potent anti-leukemic activity toward bone marrow blasts from patients with AML, including those with little or only minor responses to agents like AC220 or PKC412 [77]. G-749 was shown to induce complete elimination of leukemia cells and prolonged survival in animal models. G-749 appears to be a novel druggable candidate for the treatment of relapsed and refractory AML patients with various FLT3-ITD/FLT3-TKD mutants. G-749 may be a next-generation FLT3 inhibitor with the ability to overcome drug resistance.

#### AMG 925

AMG 925 is a highly bioavailable dual kinase inhibitor of cyclin-dependent kinase 4 (CDK4) and FLT3 and active against many FLT3 mutants reported to date [78]. AMG 925 suppresses the proliferation of tumor cell lines and exerts an anti-tumor activity by inhibiting STAT5 and RB phosphorylation. Furthermore, AMG 925 was also found to inhibit D835Y that are resistant to FLT3 inhibitors such as sorafenib and AC220 (quizartinib). In an animal model bearing AML xenograft, AMG 925 was shown to inhibit tumor growth by 96 to 99%. AMG 925 by targeting both FLT3 and CDK4 may improve clinical responses of patients with FLT3**/**ITD-mutated AML and overcome drug resistance [79].

#### TTT-3002

TTT-3002 is a novel FLT3 inhibitor with the most potent activity against a broad spectrum of FLT3-activating point mutations, including D835 and F691 L gatekeeper mutations. Compared with several other TKIs currently in clinical trials, TTT-3002 is only moderately protein-bound. TTT-3002 maintains its effect on cells isolated from patients with relapsed AML that are resistant to sorafenib and AC220. Tumor burden in an FLT3 TKI-resistant transplant mouse model was significantly reduced by oral administration of TTT-3002 [80]. TTT-3002 is cytotoxic to leukemic blasts isolated from FLT3/ITD-expressing AML patients while displaying minimal toxicity to normal hematopoietic stem/progenitor cells from healthy blood and bone marrow donors [81]. Therefore, these preclinical activities of TTT-3002 may suggest that it has the potential to become a promising new generation of FLT3 TKI for FLT3-mutated AML.

#### FF-10101

FF-10101 is a novel selective and irreversible FLT3 inhibitor with activities against FLT3/ITD, MOLM-13, MOLM-14, MV4-11, D835, Y842, and F691. FF-10101 covalently binds to the cysteine residue at 695 of FLT3 kinase and was shown to have high selectivity and inhibitory activity against FLT3 kinases. It significantly suppresses the growth of 32D cells with FLT3/ITD/D835Y-or FLT3/ITD/F691 L-expressing cells and primary AML cells with FLT3-ITD or FLT3-D835 mutations both in vitro and in vivo [82]. These evidences demonstrated that FF-10101 is a promising novel FLT3 inhibitor with activities against multiple FLT3 mutations including the activation loop mutations clinically identified as quizartinib-resistant mutations.

### Overcoming resistance to FLT3 inhibitors

Many studies have shown that FLT3 inhibitors have favorable clinical activities for AML patients with FLT3/ITD, but response duration remains short because of the rapid development of resistance. Resistance to FLT3 inhibitors was attributed to the emergence of new mutations. The secondary FLT3 tyrosine kinase domain (TKD) mutation was one of the new mutations in the patients who showed resistance to FLT3 inhibitors [83, 84]. The constitutive activation of critical tyrosine residues in the FLT3 mutants and downstream signaling effectors was the common resistance mechanism of FLT3 TKIs [85].

Combining FLT3 inhibitors with other agents is the major drive in the clinical trials to overcome the resistance to current FLT3 TKIs. Dayal successfully used a collaborative FLT3 inhibitor, HSD 1169, to act against FLT3/ITD and sorafenib-resistant cell lines [86]. In a recent report, PI3K-delta inhibitor had synergistic anti-tumor activity with FLT3 inhibitors [87]. In addition, an autophagy inhibitor TAK-165 can induce cancer cell death through the activation of chaperone-mediated autophagy to enhance the efficacy of cancer therapies [88]. By integrating these novel inhibitors in combination with FLT3 inhibitors, their efficacy may be further improved in the near future. Bispecific antibodies, immune checkpoint inhibitors, and chimeric antigen receptor (CAR) T cells are major modalities of novel cancer immunotherapy [86–102]. Crenolanib was already shown to have synergistic activity with FLT3-targeted CAR T cells [67]. It is intriguing to consider integrating FLT3 inhibitors into cancer immunotherapy for enhancing activities and minimizing resistance.

## Conclusion

FLT3 inhibitors have shown promising efficacies in aggressive AML. However, the duration of clinical response is short because of the rapid development of resistance. Novel next-generation FLT3 inhibitors are in active development to concur the resistance. Combining FLT3 inhibitors with other targeted agents are additional areas of investigation to minimize resistance to current FLT3 inhibitors.

## Abbreviations

allo-HSCT: Allogeneic hematopoietic stem cell transplantation
ATRA: All-trans retinoic acid
CR: Complete remission
CRi: Complete remission with incomplete count recovery
CRp: Complete remission with incomplete platelet recovery
DFS: Disease-free survival
EFS: Event-free survival
HSCT: Hematopoietic stem cell transplantation
LFS: Leukemia-free survival
OR: Overall response
OS: Overall survival
PD: Progress disease
PFS: Progression-free survival
RFS: Relapse-free survival
SD: Stable disease
